# A mixed-methods evaluation of the indoor and outdoor smoking ban in dining venues in Armenia: Early successes and challenges

**DOI:** 10.18332/tid/174899

**Published:** 2023-12-14

**Authors:** Zaruhi Grigoryan, Zhanna Sargsyan, Diana Muradyan, Kristina Mnatsakanyan, Varduhi Hayrumyan, Varduhi Petrosyan

**Affiliations:** 1 Turpanjian College of Health Sciences, American University of Armenia, Yerevan, Armenia

**Keywords:** policy, smoke-free, denormalization of tobacco use, challenges of policy implementation, benefits of smoking ban

## Abstract

**INTRODUCTION:**

Since March 2022, Armenia introduced a comprehensive smoking ban on all types of tobacco products in indoor and outdoor areas of hospitality venues. We aimed to rapidly appraise the implementation of the ban in the dining areas of the capital Yerevan and explore any differences in compliance and enforcement patterns between indoor and outdoor areas of the venues.

**METHODS:**

We used a mixed-methods approach through quantitative air quality monitoring, qualitative observations, and in-depth interviews (IDIs). We visited one venue in each remote district of the city and more venues from the central districts that have a much higher density of dining areas. Overall, we made 24 measurements of PM_2.5_ particles, 24 unobtrusive observations in the 19 visited venues, and 11 IDIs with six visitors and five workers. We used Stata13 for the analysis of numerical data and completed direct deductive content analysis of the textual data.

**RESULTS:**

Active tobacco use was observed in 12 out of 24 venues (50.0%) with more cases of smoking in outdoor areas (10 out of 12; 83.3%). No warning by workers or no reports to the police were observed. We detected elevated levels of PM_2.5_ particles in indoor and outdoor areas. The IDIs revealed predominantly negative attitudes towards the outdoor ban and the lack of awareness of and readiness to engage in the enforcement measures. The lack of enforcement by the owners and the respective bodies was mentioned as a contributor to continued violations of the ban. The change in the dynamic and the characteristics of the visitors, cleaner air, and less unpleasant work were mentioned as important positive aftermaths of the ban.

**CONCLUSIONS:**

The Government of Armenia should enhance the monitoring and enforcement activities and organize tailored awareness-raising campaigns to inform the general public and the hospitality industry of the health and social implications of the ban.

## INTRODUCTION

Tobacco use remains one of the major contributors to preventable morbidity and mortality worldwide^1^. Despite the existing evidence-based tobacco control strategies and corresponding national policies, tobacco use and secondhand smoke (SHS) exposure still kill over eight million people around the world yearly^1^. Conclusive evidence suggests that there is no safe level of SHS. Hence, only complete smoking bans can prevent the harm of SHS, promote quitting, and prevent initiation of tobacco use^2^. Currently, 1.8 billion people across 67 countries are covered with complete indoor smoke-free regulations which make the protection of people from tobacco smoke the second mostly adopted MPOWER measure worldwide^3^. However, there are 128 countries that still lag behind with minimal, partial or no smoking bans whatsoever^3^, and only in 14 countries high compliance with existing smoke-free regulations has been observed^4^. An extension of the smoking bans over outdoor areas is a reasonable opportunity to strengthen tobacco control efforts and denormalize the behavior^5^.

Armenia has been facing one of the highest adult male smoking rates in the European Region, 53.2% in 2022^6^. The recent national survey showed that in the past 30 days 45.1% of respondents have been exposed to SHS in closed public places including cafés, schools, and shops whereas in open public places including parking areas, sports venues, and parks, the overall SHS exposure for the same period reached 73.9%^6^.

Armenia was the first post-Soviet country that ratified the Framework Convention of Tobacco Control (FCTC) in 2004 and subsequently developed the first national tobacco control law enacted in 2005. Along with numerous provisions, the law included smoking bans in cultural, educational and healthcare facilities, and in public transportation^7^. However, the inadequate enforcement of the law during the preceding years was documented^8^. Only in 2020, the new national tobacco control law that was harmonized with the (FCTC) instituted a comprehensive smoking ban^9^. It came into force on 15 March 2022, and banned the use of all types of tobacco products (e.g. conventional cigarettes, heated tobacco products, e-cigarettes, hookahs, etc.) in indoor and outdoor areas of hospitality venues (canteens, restaurants, cafés, bars, buffets, etc.). The law also required the hospitality venues to display ‘No smoking’ and penalty signs. The supervision of the ban is designated to the police. In case of detected smoking in the banned areas, a fine of 50000 AMD (about 120 US$) is assumed for the individuals and 150000–200000 AMD (about 375–500 US$) for business entities. The implementation of the law was not accompanied with a large-scale awareness-raising campaign; however, before the law was passed it was widely covered in the mass media through press coverages, TV reportages, and interviews with health experts and authorities. Furthermore, the Government of Armenia initiated a few meetings with stakeholders, e.g. hospitality and tourism industry, to orient into new smoke-free environment at the hospitality venues. Topuridze et al.^10^ examined the support for the smoke-free policies among the general Armenian population before the implementation of the smoking ban in Armenia in 2018 and found high-level of support for smoke-free policies in public places such as healthcare, religious, governmental, and workplace settings, but relatively lower support for the smoke-free policies in outdoor areas of cafes and restaurants. Following the introduction of smoking restrictions, continuous monitoring of compliance is key for appraising the situation, tracking the progress, and uncovering existing obstacles^11^. The evaluations of smoking ban implementation in various jurisdictions have shown that poor compliance was linked to suboptimal penalization for the violations, insufficient support from the venue owners, and prevailing norms around tobacco use. Poor knowledge and inadequate attitudes were also shown to fuel the non-compliance both among the workers and the visitors^12^. Six months into the implementation of the smoking ban in hospitality venues in Armenia, we wanted to rapidly appraise the situation around its implementation and explore any differences in indoor and outdoor areas.

## METHODS

### Study design

The assessment used a mixed-methods approach through quantitative air quality monitoring and qualitative observational research techniques and in-depth interviews (IDIs). The literature suggests various dimensions and methods for assessing the implementation of smoke-free policies^13^. Since burning tobacco generates high levels of fine particles with a mean aerodynamic diameter of 2.5 μm (PM_2.5_) in indoor and outdoor spaces, it is used as a proxy measure for SHS during air quality monitoring. The WHO recommends 25 μg/m^3^ 24-hour mean concentration of PM_2.5_ for outdoor environments^14^. Observations of hospitality venues provide factual information on the environment and behaviors when smoking occurs and provide the necessary type of data to quantify the level of enforcement to inform policymakers. Thus, it is a reliable and valid measure of compliance^13^. Interviews with hospitality sector workers and visitors provide deep insights about perceptions and practices about the smoke-free policy implementation and reveal reasons for non-compliance and related challenges^13^.

### Study setting

From the hospitality industry, we focused on dining venues only and specifically on cafés and restaurants that either had both indoor and outdoor areas or only an indoor or an outdoor area. If there were no visitors in the selected venue, the research team skipped and approached the next venue in the list. To explore the diverse experiences throughout the city, the study team attempted to visit one busy venue in each of the 11 districts of Yerevan (the capital city) with more venues from the central ones, where the majority of the dining venues are located. However, we ended up visiting cafés and restaurants only in eight districts, as no eligible dining venues were identified in three peripheral districts. As a result, we visited more than one venue in districts that had a higher density of venues.

### Study participants

The participants for IDIs were purposively and conveniently selected. We recruited participants from the researchers’ personal network. People who frequently visited dining venues and were fluent in Armenian were invited for the interviews. To gain wider perspectives and reach better generalizability and credibility of the study results, we purposively recruited participants of different age groups, gender, and smoking status, both among the workers and visitors.

### Study instrument

We developed a standardized observation form that included questions on compliance practices such as presence of tobacco use, ashtrays, smell of smoke, and cigarette butts. For the enforcement practices, we observed the presence of ‘No smoking’ and penalty signs, reporting workers’ or other visitors’ requests to put away the tobacco to the police. Additionally, the checklist contained questions to observe presence of alternative PM_2.5_ sources (candles, open fire, or grills), the locations of tobacco use (around the table or other spaces banned for smoking such as any indoor/outdoor area banned for smoking, outdoor area illegally designated for smoking, near the main entrance or at the entrance to an indoor area) (Supplementary file). The observation form was embedded into Alchemer online platform which also allowed simultaneous data entry (https://www.alchemer.com/). We also developed two semi-structured IDI guides for workers and visitors. They included open-ended questions regarding the participants’ awareness of and attitudes toward the existing smoking ban, their experience of compliance with and enforcement of the ban, reasons for non-compliance and any challenges faced since the inaction of the ban.

### Data collection

The data collection was held during September 2022. At the time of the study all COVID-19-related restrictions were lifted, including maintenance of social distancing and wearing of face masks. The study team consisted of four researchers trained in quantitative and qualitative research techniques who did the observations and air quality monitoring unobtrusively so as not to intervene in the natural behaviors^15^. Overall, we did 24 observations and 24 air quality monitoring sessions in 12 indoor and 12 outdoor areas in the visited 19 venues of which five had both indoor and outdoor areas. Data collection was done following a schedule of two visits per day (during 6–10 p.m.). We used a laser photometer that assesses the real-time concentrations of PM_2.5_ particles (TSI SidePak AM520 Personal Aerosol Monitor; TSI Incorporated, Minnesota, USA). We used a standard protocol for the measurements in indoor and outdoor areas; cleaned the inlet filter of the device daily, as per the factory recommendations, and used a calibration factor of 0.3 as suggested for the measurements of the SHS^16^. Each air quality monitoring session lasted at least 30 minutes. Since the tobacco-related PM_2.5_ concentration decreases by half in 55 minutes (median time), we would expect the device to capture even some elevated levels of PM_2.5_ because of smoking that occurred before the observation^17^. The data was logged at 1-minute intervals. The device was placed in a shoulder bag on the level of a table, and, in case of active smoking, the team ensured a 1 m distance from the smoker^18^. For each observed area, distinct PM_2.5_ measurements were done.

We conducted 11 IDIs with five workers and six visitors with a duration varying from 15 to 30 minutes. The IDIs were audio-recorded upon receiving the participants’ permission. Otherwise, detailed notes were taken during the interviews. The interviews were stopped upon reaching data and meaning saturation.

### Data management and analysis

Observation data were exported from the Alchemer, and air quality monitoring data were backed up in a computer using TrakPro5 software, daily. We used Stata13 and TrakPro5 for the analysis. Descriptive statistics were used to describe geometric mean (GM) and median values of the PM_2.5_ particles. Two-tailed, independent samples Student’s t-test and Pearson’s chi-squared test were used to compare the data on the measured PM_2.5_ concentrations (log-transformed), and smoking practices in indoor versus outdoor areas at 0.05 significance level.

The qualitative interviews were transcribed in Armenian verbatim and direct deductive content analysis of the data was done following the domains of the interview guides^19^. The findings were categorized under the three themes: 1) Awareness of and attitude towards the smoking ban, 2) Implementation of the ban, and 3) How to strengthen compliance – opportunities for improvement. The first theme covers the participants’ awareness of the smoking ban in dining venues, their attitudes about the indoor and outdoor restrictions as well as awareness and opinions about the enforcement mechanisms. The second theme presents two categories of findings such as visitors’ observations and workers’ experiences about the enforcement of the ban and engagement of visitors as members of the general community in facilitating the enforcement. The third theme presents the main recommendations by the participants for strengthening the implementation of the ban.

## RESULTS

### Observations and air quality monitoring

**Figure 1:**
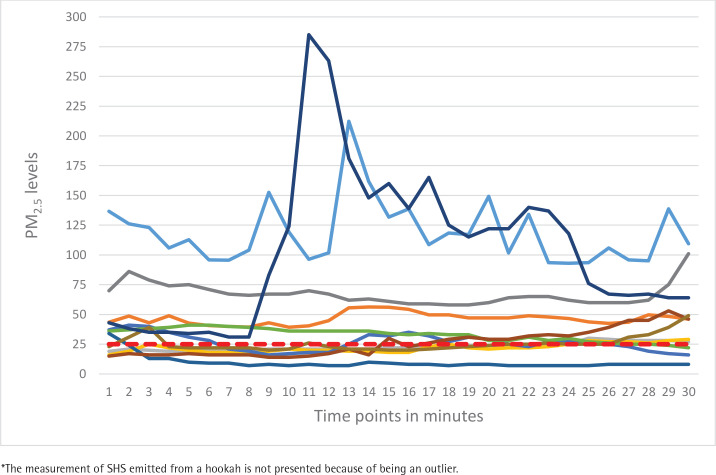
Detected PM_2.5_ concentrations (μg/m^3^) in indoor areas of dining venues, Yerevan, 2022 (N=11)*

**Figure 2:**
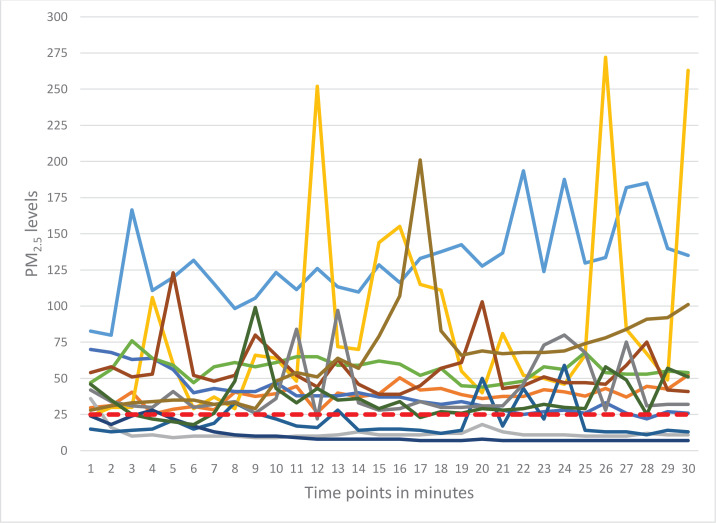
Detected PM_2.5_ concentrations (μg/m^3^) in outdoor areas of dining venues, Yerevan, 2022 (N=12)

**Figure 3:**
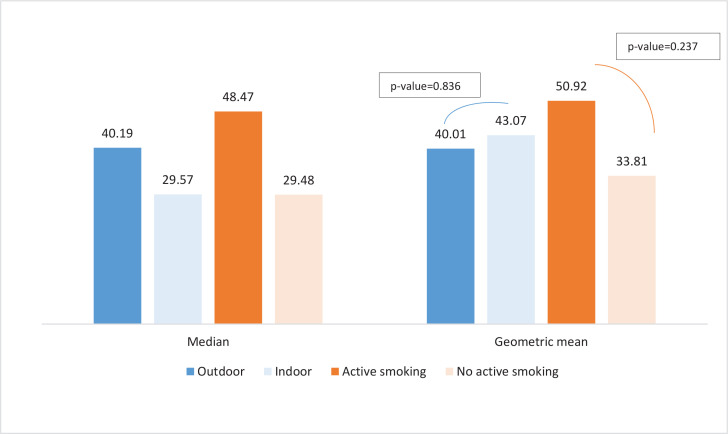
Detected PM_2.5_ concentrations (μg/m^3^) in indoor and outdoor areas of dining venues, by venue type and observed/non-observed active smoking, Yerevan, 2022 (N=24)

Active tobacco use was detected in 50.0% of the observations with more cases of smoking detected in outdoor areas (83.3%; n=10) (Table 1). Tobacco use was mostly observed around the tables (83.3%). In nearly 42% of observations, ashtrays were present on the tables or were brought by the workers upon the visitors’ requests. The proofs of tobacco use such as cigarette butts (41.7%) and the smell of smoke (83.3%) were more commonly observed in outdoor areas. The differences in observed active smoking, cigarette butts, and smell of smoke between outdoor and indoor areas were statistically significant. No tobacco advertising was observed in the visited venues. ‘No smoking’ and penalty signs were observed only in seven and two venues, respectively. Despite the observed active smoking, no warning to put away tobacco or no reports to the police were observed. The mean number of smokers was higher in the outdoor areas (p=0.023) and in the majority of observations (91.7%) the smokers were men (Table 2). Air quality monitoring detected elevated levels of PM_2.5_ particles both indoors and outdoors (Table 3 and Figure 1). The GM concentration of PM_2.5_ particles in the total sample was 41.49 μg/m^3^. On average, this exceeds the WHO-recommended threshold of 25 μg/m^3^ by 1.7 times. The GM concentrations in areas where active smoking was observed was 1.5 times higher than in venues where no active smoking was observed (50.92 μg/m^3^ vs 33.81 μg/m^3^). The maximum level of PM_2.5_ was detected in an indoor area of a hookah bar and reached 1620 μg/m^3^, 60 times higher than the recommended threshold. Figures 2 and 3 present the 30-minute air quality monitoring measurements in 11 indoor (excluding the outlying measurement where SHS from hookah was captured) and 12 outdoor areas, respectively.

**Table 1:** Observed compliance with and enforcement of the smoking ban in indoor and outdoor areas of dining venues, Yerevan, 2022 (N=24)

*Compliance and Enforcement*	*Total (N=24) % (n)*	*Indoor (N=12) % (n)*	*Outdoor (N=12) % (n)*	p*
**Compliance**				
Active tobacco use	50.0 (12)	16.7 (2)	83.3 (10)	**0.001**
**Locations of active tobacco use**				
Around the table	83.3 (10)	16.7 (2)	66.7 (8)	
Other spaces banned for smoking	16.7 (2)	-	16.7 (2)	
Ashtrays on the tables or brought upon request	41.7 (10)	25.0 (3)	58.3 (7)	**0.012**
Cigarette butts	20.8 (5)	0	41.7 (5)	**0.012**
Smell of smoke*	54.2 (13)	25.0 (3)	83.3 (10)	**0.004**
Alternative PM_2.5_ sources	16.7 (4)	33.3 (4)	0	
Tobacco advertising	0	-	-	
**Enforcement measures**				
‘No smoking’ signs	29.7 (7)	33.3 (4)	25.0 (3)	0.653
Penalty signs	8.3 (2)	8.3 (1)	8.3 (1)	1.000
Report to the police	0	-	-	
**Request to put away tobacco**	0			
By workers	-	-	-	
By visitors	-			
**Designated area for tobacco use**	0	-	-	

* Pearson’s chi-squared test.

**Table 2:** Characteristics of tobacco use in indoor and outdoor areas where active smoking was observed, Yerevan, 2022 (N=12)

*Characteristics*	*Total (N=12) % (n)*	*Indoor (N=2) % (n)*	*Outdoor (N=10) % (n)*	p*
**Number of smokers^a^,** mean % of smokers (SD)	13.2 (2.52)	23.5 (0.33)	1.7 (4.72)	**0.023**
**Type of consumed tobacco products (n=12)**				
Conventional cigarettes	91.7 (11)	50.0 (1)	83.3 (10)	**0.020**
E-cigarettes	33.3 (4)	-	40.0 (4)	0.273
Heated tobacco products	8.3 (1)	-	10.0 (1)	0.640
Waterpipe	16.7 (2)	50.0 (1)	10.0 (1)	0.166
**Approximate age of smokers** (years)				
19–35	58.3 (7)	50.0 (1)	60.0 (6)	0.793
36–50	58.3 (7)	50.0 (1)	60.0 (6)	0.793
51–65	33.3 (4)	0	40.0 (4)	0.273
**Gender of smokers**				
Male	91.7 (11)	100.0 (2)	90.0 (9)	0.640
Female	8.33 (1)	0	10.0 (1)	
**Pattern of tobacco use** (n=12)				
Group	66.7 (8)	1	70.0 (7)	0.273
Individual	66.7 (8)	100.0 (2)	60.0 (60)	0.584

* Two-tailed independent samples Student’s t-test or Pearson’s chi-squared test.

a Number of smokers represents the ratio of the mean smokers and the mean of visitors in the total sample (19.1), indoor (18.1), and outdoor areas (20.0).

**Table 3:** Detected PM_2.5_ concentrations (μg/m^3^) in indoor and outdoor areas of dining venues, by venue type and observed/non-observed active smoking, Yerevan, 2022 (N=24)

*Detected PM_2.5_ concentrations (μg/m^3^)*	*Total (N=24)*	*Indoor (N=12)*	*Outdoor (N=12)*	p*	*Active smoking (N=12)*	*No active smoking (N=12)*	p*
Median (IQR)	38.68 (24.68–66.72)	29.57 (24.69–86.49)	40.19 (28.71–61.16)	-	48.47 (32.08–76.44)	29.48 (22.83–57.14)	-
Geometric mean (95% CI)	41.49 (29.17–59.01)	43.07 (23.29–79.52)	40.01 (25.33–63.20)	0.836	50.92 (29.04–89.28)	33.81 (20.82–54.90)	0.237

IQR: interquartile range.

* Two-tailed independent samples Student’s t-test using log-transformed data.

### Theme 1: Awareness of and attitude toward the smoking ban

*Awareness of and attitudes toward the indoor and outdoor smoking restrictions*

All respondents mentioned that they are aware of the new smoking ban. The majority favored the ban and highlighted its potential positive impact such as prevention of smoking among the youth and protection of non-smokers:

*‘I support the ban. If my child sees that her parents do not smoke, I am sure s/he will not smoke in the future.’* (Visitor 1, non-smoker, female)

*‘The purpose of the ban is to prevent the loss caused by tobacco use as its smoke harms others’ health.’* (Worker 3, smoker, male)

However, almost half of the participants disputed the outdoor ban stating that the indoor ban only would have been enough or that the designation of separate areas or special venues for smokers would be a better option:

*‘The indoor smoking ban is a good one as closed areas are not properly ventilated, whereas in outdoor areas there is no such issue … every pedestrian can smoke while walking near the café and the visitors will still be exposed to that smoke.’* (Worker 1, non-smoker, male)

*‘There could be special venues where smoking would be allowed. In that case, the law would have been more adequate.’* (Worker 6, non-smoker, male)

*Awareness of and attitudes toward the enforcement measures*

In contrast to the workers, the majority of visitors did not know what actions should be followed in case of noticing a violation of the ban:

*‘I do not know what I am supposed to do in case of noticing a smoker.’* (Visitor 1, non-smoker, female)

The workers stressed that the fines are a necessary factor for implementing the ban also acknowledging that effective enforcement is difficult without consistent penalization of violations:

*‘I agree that a fine should be applied. It will have an effect because people will start respecting the law and will smoke only wherever allowed.’* (Worker 1, non-smoker, male)

Most of the participants were against reporting the violation to the police stating that it is an inappropriate, ‘shameful’, or irrelevant way for enforcing the smoking ban:

*‘Our population's mentality will not allow it [reporting to the police]. Probably they will do that only in extreme situations.’* (Visitor 2, smoker, male)

*‘I think if I call the police and say that a person is smoking here, the police officer will make fun of me … it is not relevant to call the police officer for this.’* (Worker 3, smoker, male)

### Theme 2: Implementation of the ban

*Enforcement of the smoking ban by workers*

The majority of workers mentioned not having any problem with enforcing the ban in indoor areas. According to them, most of their visitors do not try to smoke, but even those who attempt to smoke immediately put away the cigarette after a warning:

*‘When we say that "you can’t smoke"’, believe me, it helps.’* (Worker 4, non-smoker, female)

However, the workers’ experience of enforcing the smoking ban outdoors was more challenging. They specified that some visitors get frustrated because of it as they are still able to smoke in other outdoor venues. Nevertheless, the workers concluded that their visitors eventually put up with the ban and comply with it:

*‘We politely ask them not to smoke outdoors. Sometimes, they get frustrated and bring examples of other cafés where outdoor smoking is permitted …’* (Worker 1, non-smoker, male)

Almost all visitors stated that the workers of the venues they visit remind visitors about the ban and, if necessary, kindly ask the smokers to put away the tobacco or to smoke outside of the venue:

*‘Workers warn visitors about the smoking ban in indoor areas. Whenever a visitor asked if he could smoke in an indoor area, the worker kindly asked him/her to go out to smoke.’* (Visitor 4, non-smoker, female)

The removal of ashtrays from the table was the most common enforcement measure as mentioned by the workers, however, some workers mentioned bringing ashtrays upon request in outdoor venues:

*‘The owner of the venue ordered not to put ashtrays on the tables either in indoor or outdoor areas. But when the visitors ask for an ashtray, we give it to them.’* (Worker 4, non-smoker, female)

*‘Currently, when the law is in force, people smoke only hookah in indoor and outdoor areas. But in the outdoor area, we have a place where we allow the visitors to smoke a cigarette.’* (Worker 6, smoker, male)

*Compliance with the smoking ban by visitors*

The workers and visitors mentioned that they still notice active smoking or attempt to smoke in banned areas. Some workers also reflected on shockingly non-compliant visitors who do not respond to the warnings or react improperly, i.e. agree to pay the fine and continue smoking or leave the venue without paying the bill:

*‘I saw a smoker in the café but nobody noticed it and the visitor continued smoking. I saw him but I also did not say anything.’* (Visitor 4, non-smoker, female)

*‘In many places people still smoke in outdoor areas.’* (Worker 4, non-smoker, female)

*‘Once we ask the visitor to stop smoking and informed about the fine, and he said that he is ready to pay the fine but continued to smoke.’* (Worker 3, smoker, male)

*‘One of our workers kindly asked a visitor not to smoke. The visitor went out without paying the bill.’* (Worker 2, non-smoker, female)

Interestingly, the visitors mentioned that they would rather tolerate the smoke unless it disturbs them:

*‘Only if I feel that the smoke irritates my eyes I will ask the smoker to stop. Otherwise, I think I will do nothing.’* (Visitor 1, smoker, male)

However, the participants mentioned that though in the beginning many visitors were aggressive towards the ban, they gradually got used to it:

*‘When the ban was just introduced, other [smoker] visitors were frustrated about going out to smoke, but then they got used to it.’* (Visitor 6, non-smoker, female)

### Theme 3: How to strengthen the implementation – opportunities for improvement

The participants outlined several shortages that need to be addressed to improve compliance. First, they pointed out the lack of coordination and administrative support for exposing and addressing non-compliance. According to them, the suboptimal oversight and enforcement of the ban resulted in continued smoking in dining venues:

*‘Everyone should be consistent with the law. The government should monitor and evaluate how the law works always, and not only in the first two months [of inaction].’* (Worker 4, non-smoker, female)

Additionally, the participants mentioned that the venue owners’ proper support for the enforcement and more awareness-raising activities are needed:

*‘The owners of the venues should be stricter and take corresponding measures to prohibit smoking.’* (Visitor 6, non-smoker, female)

*‘The mass media, television, radio as well as posters should all state that tobacco harms our health and causes many diseases.’* (Worker 5, non-smoker, male)

Though some visitors and workers expressed concerns over presumable ‘negative impact’ of the ban on the hospitality industry, most of the workers, listed rather positive aftermaths of the ban. Particularly workers noticed a change in the dynamic and the characteristics of their visitors. The workers were also glad that they no longer have to empty the ashtrays and that their health will not suffer because of the exposure to SHS:

*‘I had a fear that people would not visit cafés, but the ban was quickly accepted and did not cause any similar effects.’* (Worker 3, smoker, male)

*‘In cold weather male visitors used to come and order just coffee and water and stay for hours… it was very irritating as the workers had to empty the ashtrays. Now, idle people do not visit our venue.’* (Worker 2, non-smoker, female)

*‘One of the positive consequences is that women come with children and families more often and, now, our venue is like a family restaurant ... also the health of the staff is not harmed.’* (Worker 1, non-smoker, male)

## DISCUSSION

This work draws on the findings of a study that examined the situation around the implementation of the smoking ban in dining venues in Armenia through objective measurements and exploring the perspectives and experiences of workers and visitors. Our study found an inadequate implementation of the smoking ban due to ineffective enforcement efforts, lack of compliance, and lack of support for the ban.

The majority of participants were aware of the ban, yet not all were properly informed about the enforcement measures. The overarching complaint of the participants, regardless of the smoking status, was that the smoking ban should be applied mainly to indoor areas. This indicates that the participants failed to acknowledge the social impact that the outdoor ban can bring by denormalizing smoking and preventing the harm of SHS. Numerous studies have discussed the impact of smoke-free legislation on the public’s attitudes which extended to social unacceptability of smoking, established smoke-free role models for the youth, and increased quitting or intentions to quit^20,21^. These studies also acknowledged that the shifts in smoking-related social norms require time to occur^21,22^. Consistency in the enforcement and awareness-raising activities would be crucial for overcoming the prevailing attitudes surrounding smoking in Armenia. The evidence on smoke-free policy implementation shows that comprehensive bans result in better compliance and are easier to enforce^23^. Moreover, an unattended disobedience with the smoking ban could worsen the existing level of compliance, and, thus, undermine the attained success, especially in indoor areas of dining venues in Armenia^24^. The detected high levels of PM_2.5_ particles in venues where the use of hookahs was observed, warn that the smoking ban is not comprehensively implemented against all types of tobacco products, which, if left unaddressed, may also undermine the implementation of the ban in Armenia.

The study showed tolerance towards outdoor smoking in Yerevan. Air quality monitoring also detected elevated levels of PM_2.5_ particles both indoors and outdoors; however, lower concentrations of PM_2.5_ particles in outdoor areas are potentially due to air movements that dilute the SHS^25^. Despite the small sample of visited venues, some of the differences between outdoor and indoor areas were statistically significant: more active smoking, cigarette butts, and smell of smoke were observed outdoors.

With regard to the enforcement efforts, we found that workers did not warn the tobacco users outside to stop smoking, and did not report violations to the police either because of not trusting that police engagement was effective or because of thinking the measure was not appropriate. This finding suggests that the enforcement measure is not yet operational in Armenia and targeted actions are needed to overcome such barriers or alternative enforcement measures should be considered. Various countries have delegated the enforcement of the provisions of tobacco control laws to police, and the reported experiences are diverse. Studies in the US, India, and Kenya suggest that the police do not prioritize enforcement of tobacco control, they were already overloaded to meaningfully contribute to it, or they were not skilled to do the job^26-28^. In contrast, compliance with smoke-free policies improved after involvement of trained police officers in Vietnam^29^.

Our study also revealed lack of proper monitoring of the ban and applying fines. The international evidence is also clear that fines are among the most important factors for successful policy enforcement and the lack of fines is listed among barriers^30^. Montini et al.^31^ found that specific venues are more likely to comply with smoke-free regulations if other venues in the area are also compliant. Thus, proper enforcement has the potential of a multiplicative effect in terms of increasing compliance with the ban; whereas, non-compliance can lead to the opposite effect.

The study participants reported reliance solely on the Government for the enforcement indicating lack of community participation in creation of safer smoke-free environments. Numerous studies highlight the importance of active engagement of main actors including the community members, workers, smokers, and the governments for ensuring the successful implementation of laws^32,33^. Assuring public participation in the smoking ban enforcement in Armenia through tailored awareness-raising efforts can contribute to breaking the dominant social norm of tolerating smoking^34^.

Lastly, the arguments about the negative impact of the ban on the hospitality industry raised by a couple of participants were not largely considered as an issue by the majority. Indeed, numerous studies have found that smoking bans have no negative economic impact on businesses^35^. Yet, for over a decade the tobacco industry has fueled such false claims which have been widely accepted by the hospitality sector and have created difficulties for tobacco control activities^36^. In our study, workers identified various positive aftermaths of the ban including the change in the dynamic and the characteristics of the visitors, cleaner air, and less unpleasant work for them that could serve as opportunities to further motivate the workers of the hospitality industry to enforce the law.

### Limitations

Our study has several strengths and limitations. With the mixed-methods approach, we triangulated the findings between various sources and data collection methods, which gave a clearer picture of the compliance level with and the enforcement of the smoking ban in Armenia. This was the first rapid appraisal of the smoking ban implementation, which revealed existing challenges and early successes since the inaction as well as unique experiences of indoor and outdoor smoking ban enforcement. The small sample of the visited venues located in Yerevan did not allow us to make inferences about the situation in the country, though the compliance might be poorer in distant cities. Additionally, the situation around compliance in indoor areas might change with seasonal changes, especially during cold weather. The venue selection process was prone to selection bias. Additionally, due to small sample size, we were limited to only descriptive comparisons of PM_2.5_ levels across venue types and smoking practices, and no adjusted analysis was done.

## CONCLUSIONS

Given the poorer situation concerning the implementation of the outdoor smoking ban, targeted activities are needed to facilitate enforcement and compliance. First, the Government of Armenia should enhance the monitoring and enforcement activities. Second, targeted and tailored awareness-raising activities are needed both for the general public and the hospitality industry. The general public should be informed about the specific features as well as the health and social implications of the ban and specifically their role in denormalizing tobacco use.

The findings of this study might be relevant for other countries that face a similar tobacco use burden and similar issues. Armenia’s experience, early accomplishments, and challenges might inform the efforts of other countries that are on the way to implementing comprehensive indoor and outdoor smoke-free policies in public places.

## Data Availability

The data supporting this research are available from the authors on reasonable request.
