# Supplementary material for: A mixed-methods evaluation of the indoor and outdoor smoking ban in dining venues in Armenia: Early successes and challenges
Source: Tob Induc Dis. 2023 Dec 14;21:167. doi: 10.18332/tid/174899 (PMC10720263; doi:10.18332/tid/174899)
Supplement: Supplementary file 1 [file TID-21-167-s1.pdf]

SUPPLEMENTARY MATERIALS

Appendix 1: Interview Guide for workers of dining venues

Thank you very much for agreeing to participate in this study. Let's start the interview by talking about your experience of working in this venue:

- How long have you been working in this café/restaurant?
- How do you feel about being exposed to secondhand smoke while working in this venue before the smoke-free legislation in hospitality venues entered into force? And what about now?

Now let's go into details about the smoking ban in hospitality venues and your knowledge and experience regarding that ban.

I. Knowledge of the smoke-free law

1) What do you know about the new smoking ban that was enacted starting March 15, 2022? Can you describe the ban and its purpose (establish and maintain smoke-free environments)? How did you learn about it?

II. Attitude of the smoke-free law

1) What do you, generally, think about the new smoking ban in indoor and outdoor areas of the hospitality venues?
2) What do you think, is having smoking ban in indoor and outdoor areas important?
3) What do you think are those ban voluntary or mandatory? Please explain. In what situation it is mandatory and in what situation it is voluntary? What are the advantages and disadvantages of mandatory smoking ban?
4) Please comment on whether it is too strict or it is reasonable to have both indoor and outdoor smoking bans. Please elaborate.
5) What are/will be some potential positive effects of indoor and outdoor smoking ban in your hospitality venue?
    - Health of staff, clean air, smoking vs non-smoking visitors, more or fewer visitors?

III. Practice: Enforcement of the law by the workers

6) Aside from the national law, what is the local smoking policy in your venue?
7) What is your experience of enforcing the law? How did your working behavior change after the new provision entered into force? Please specify, what kind of action(s) have you taken to enforce the smoke-free law in your venue?
    - Asked the visitors not to smoke
    - Did not smoke yourself/quit smoking/changed smoking practice at home
    - Collected all the ashtrays from the venue tables?
    - Posted "No smoking" sign

- Designated an area for smoking
- What would you like to do to enforce the implementation of the law?

8) How those actions differed between indoor and outdoor areas of the venue? ***If there is a difference,*** then what is the reason?
9) Please describe the current indoor and outdoor smoking real practice in your venue. Are you successful with the implementation of the law?
    - Have you noticed any active smoking in the outdoor area? What about indoor area?
    - Have you noticed any attempt for smoking in your venue?

IF NO, GO TO Q13

10) What actions followed by you or your colleagues after noticing active smoking or an attempt to smoke by a visitor?
11) How the visitors reacted?
    - Did the guest stop smoking after your notice or not?
    - What did you do when the guest did not agree to stop smoking even after you gave them a notice?
    - What is the difference between indoor and outdoor areas? And what is the reason?
    - Do you know what you have to do in that scenario as per the law?
12) What type of tobacco products are being used in indoor areas? What about outdoor areas?

13) If you ever see a guest who is smoking, what will you do? Would you approach them and ask them to stop smoking?
    - What would you do if the guest does not agree to stop smoking even after you gave them a notice?
    - Do you know what you have to do in that scenario as per the law?

14) In case if any of your visitors ask you for ashtray, would you bring one for them? Please elaborate why yes or no.

IV. Practice: compliance by the visitors

15) Since the law has been implemented, what kind of changes in smoking practices have you noticed in your visitors? Was the new policy easily accepted by them?
16) Do the visitors of your venue comply with the law? In your opinion, what are the reasons that the visitors comply with the new smoke-free law? *For example, because your venue is 100% smoke-free? What else?* And what are the factors that challenge their compliance?
17) Could you also tell what can help the visitors to be more compliant with the smoke-free law?
    - Any external factor such as financial penalty or signage of "smoke-free area"?
    - Please name any other factors that can assist implementation of the law.

V. Suggestions on how to improve the enforcement

18) Based on your recent experience, what are the factors that help the workers to enforce the new smoke-free law? Could you please also name the factors that challenge the workers in enforcing the new smoking ban?

19) Now please name the factors that can further motivate the workers or help them to more effectively implement the enforcement of the law?

20) What do you think about calling police and notifying about violation in your venue? What do you think about the financial penalty as a mean for enforcement of the smoking ban? Are those effective? Have you ever turned to those means for supporting the enforcement? Why yes or why not?

21) Do you have any suggestions on how to improve the current situation with the enforcement of the smoke-free law?

Appendix 2: Interview Guide for visitors of hospitality sector

Thank you very much for agreeing to participate in this study. Let's start the interview by talking about your interest regarding this venue:

- How often do you come to this café/restaurant?
- What do you specifically love about this place?
- Do you usually come here with your friends/ family or alone?
- When you come to this café/restaurant how long do you approximately stay here?

Now let's go into details about the smoking ban in hospitality venues and your knowledge and experience regarding that ban.

VI. Knowledge of the smoke-free law

1. Could you tell me what do you know about the new smoking ban that was enacted starting March 15, 2022? Can you describe the ban and its purpose (establish and maintain smoke-free environments, improve health of workers and visitors)? How did you learn about it?

VII. Attitude of the smoke-free law

2. What do you, generally, think about the new smoking ban in indoor and outdoor areas of the hospitality venues?
3. What do you think, is having smoking ban in indoor and outdoor areas important?

4. What do you think are those bans voluntary or mandatory? Please explain. In what situation it is mandatory and in what situation it is voluntary? What are the advantages and disadvantages of mandatory smoking bans?
5. Please comment on whether it is too strict or it is reasonable to have both indoor and outdoor smoking bans. Please elaborate.
6. What are/will be some potential positive effects of indoor and outdoor smoking bans in your hospitality venue?
   a. Health of staff
   b. Clean air
   c. Smoking vs non-smoking visitors

VIII. Practice: Enforcement of the law by the workers

7. Have you noticed any change in workers' behavior in terms of enforcing the law in hospitality venues? Please specify.

IX. Their attitudes towards the protective nature of the law

8. Since the law has been implemented, what kind of changes in smoking practices have you noticed in other visitors? What about decrease in the use of tobacco products - have you noticed such trend?

9. Have you noticed any active smoking or smoking attempt in banned areas?
10. What actions followed by you or your colleagues after noticing active smoking or an attempt to smoke by a visitor? Have you ever asked other visitor to stop smoking? How the visitors reacted?
    - Did the guest stop smoking after your notice or not?
    - What did you do when the guest did not agree to stop smoking even after you gave them a notice?
    - What is the difference between indoor and outdoor areas? And what is the reason?
    - Do you know what you have to do in that scenario as per the law?
11. What type of tobacco products are mostly being used in indoor areas? What about outdoor areas?
12. Have you ever noticed a worker to bring an ashtray to visitors per their request?
13. What would you do in case of noticing an ashtray on the table or smell of smoke in hospitality venue?
14. If you ever see a guest who is smoking, what will you do? Would you approach them and ask them to stop smoking?
    - What would you do if the guest does not agree to stop smoking even after you gave them a notice?

- Do you know what you have to do in that scenario as per the law?

X. Suggestions on how to improve the enforcement

15. What are the factors that help the workers to enforce the new smoke-free law? Could you please also name the factors that challenge the workers in enforcing the new smoking ban?
16. Can you name factors that can help other visitors to become more complaint with the smoking ban?
    a. Any external factors such as penalty or postage of "No smoking" sign.
    b. Please name other factors, that can help the implementation of the law.
17. Now please name the factors that can further motivate the workers and other visitors to more effectively implement the enforcement of the law?
18. What do you think about calling police and notifying about violation in your venue? What do you think about the financial penalty as a mean for enforcement of the smoking ban? Are those measures effective? Have you ever turned to those means for supporting the enforcement? Why yes or why not? What can be done in this regard?

19. Do you have any suggestions on how to improve the current situation with the enforcement of the smoke-free law?

Appendix 3: Observation checklist

1) Interviewer ID ____

2) Facility ID ____

3) Date of visit ____

4) Time of entry ____

5) Type of the hospitality venue

( ) Restaurant

( ) Café

Logic: Show/hide trigger exists.

6) Area being observed

( ) Indoor

( ) Outdoor

Logic: Hidden unless: #6 Question "Area being observed" is one of the following answers ("Indoor")

7) Room Dimensions

- Length:: ____
- Width:: ____
- Height: ____

Logic: Hidden unless: #6 Question "Area being observed" is one of the following answers ("Indoor")

8) Does the indoor area have ventilation

( ) Yes

( ) No

Logic: Hidden unless: #6 Question "Area being observed" is one of the following answers ("Indoor")

9) Are the windows, that should be open to the outside, currently open?

( ) Yes

( ) No

( ) There are no windows opening to outdoor areas

10) Does the observed area have any "No-smoking" signage?

( ) Yes

( ) No

11) Does the observed area have a penalty signage for smoking

( ) Yes

( ) No

Logic: Show/hide trigger exists.

12) Is there a designated area for smoking inside the observed area?

( ) Yes

( ) No

Logic: Hidden unless: #12 Question "Is there a designated area for smoking inside the observed area?" is one of the following answers ("Yes")

13) Is there a signage indicating that smoking is allowed in that area?

( ) Yes

( ) No

14) Are there any visible tobacco advertising sings (pictures) on the tables?

( ) Yes

( ) No

15) Are there any ashtrays on the tables?

( ) Yes

( ) No

16) Does the waitress bring an ashtray upon the request of the visitor?

( ) Yes

( ) No

( ) NA (No request was observed)

17) Are there any cigarette butts visible at the venue?

( ) Yes

( ) No

18) The number of smokers outside or at the door entrance

Dropdown menu ("0-20", "More than 20")

19) Is there any presence of smell of cigarette smoke?

( ) Yes

( ) No

Logic: Show/hide trigger exists.

20) Is there any active smoking in the observed area?

( ) Yes

( ) No

Logic: Hidden unless: #20 Question "Is there any active smoking in the observed area?" is one of the following answers ("Yes")

21) Please specify the place where active smoking is observed.

MARK ALL THAT APPLY

[ ] Around the table indoor area, please specify number of smokers:

____

[ ] Around the table outdoor area, please specify number of smokers:

____

[ ] Indoor area banned for smoking, please specify number of smokers:

____

[ ] Outdoor area banned for smoking, please specify number of smokers:

____

[ ] Outdoor area designated for smoking, please specify number of smokers:

____

[ ] Near the main entrance (beyond the cafe), please specify number of smokers:

____

[ ] At the entrance to indoor area, please specify number of smokers:

____

[ ] Other, please specify where and number of smokers:

____

Logic: Hidden unless: #20 Question "Is there any active smoking in the observed area?" is one of the following answers ("Yes")

22) Type of consumed tobacco product:

MARK ALL THAT APPLY

[ ] Cigarette, please specify the number of smokers:

____

[ ] E-cigarette, please specify the number of smokers:

____

[ ] E-cigarette, please specify the number of smokers:

____

[ ] Heated tobacco product, please specify the number of smokers:

____

[ ] Cigar, please specify the number of smokers:

____

[ ] Other, please specify what other product and the number of smokers:

____

Logic: Hidden unless: #20 Question "Is there any active smoking in the observed area?" is one of the following answers ("Yes")

23) Approximate age of smokers

MARK ALL THAT APPLY

[ ] Under 18, please specify number of smokers:

____

[ ] 19-35 years old, please specify number of smokers:

____

[ ] 36-50 years old, please specify number of smokers:

____

[ ] 51-65 years old, please specify number of smokers:

____

[ ] 66 years old and above, please specify number of smokers:

____

Logic: Hidden unless: #20 Question "Is there any active smoking in the observed area?" is one of the following answers ("Yes")

24) Gender of the smokers

( ) Male, specify number: ____

( ) Female, specify number: ____

Logic: Hidden unless: #20 Question "Is there any active smoking in the observed area?" is one of the following answers ("Yes")

25) Are the smokers individuals or in a group?

( ) Individual smoking: ____

( ) Group smoking: ____

Logic: Show/hide trigger exists. Hidden unless: #20 Question "Is there any active smoking in the observed area?" is one of the following answers ("Yes")

26) Did you notice any attempts from the serving personnel or other visitors to request to stop smoking cigarettes or using other tobacco products?

( ) Yes

( ) No

Logic: Hidden unless: #26 Question "Did you notice any attempts from the serving personnel or other visitors to request to stop smoking cigarettes or using other tobacco products?" is one of the following answers ("Yes")

27) Were the attempts successful?

( ) Yes

( ) No

Logic: Hidden unless: #20 Question "Is there any active smoking in the observed area?" is one of the following answers ("Yes")

28) Were there any financial penalties for the violator?

( ) Yes

( ) No

Logic: Hidden unless: #20 Question "Is there any active smoking in the observed area?" is one of the following answers ("Yes")

29) Was the smoking case reported to the police?

( ) Yes

( ) No

30) Are there any sources of open fire (E.g. grill, oven etc.) visible at the venue?

( ) Yes

( ) No

31) Are there any burning candles available on the table?

( ) Yes

( ) No

32) Visitors info.

	The total number of visitors	Number of smokers
0 minutes	________________________________	________________________________
15 minutes	________________________________ ______	________________________________
30 minutes	________________________________ ______	________________________________

33) Other comments and Observations? ________________________________

34) Time of exit _______________

Thank You!

© 2023 Grigoryan Z. et al.
